# Morphologic and Genotypic Characterization of *Psoroptes* Mites from Water Buffaloes in Egypt

**DOI:** 10.1371/journal.pone.0141554

**Published:** 2015-10-30

**Authors:** Said Amer, Taher Abd El Wahab, Abd El Naby Metwaly, Yaoyu Feng, Lihua Xiao

**Affiliations:** 1 Division of Foodborne, Waterborne and Environmental Diseases, National Center for Emerging and Zoonotic Infectious Diseases, Centers for Disease Control and Prevention, Atlanta, Georgia, United States of America; 2 Department of Zoology, Faculty of Science, Kafr El sheikh University, Kafr El Sheikh, Egypt; 3 Animal Health Research Institute, Kafr El Sheikh Provisional Laboratory, Kafr El Sheikh, Egypt; 4 State Environmental Protection Key Laboratory of Environmental Risk Assessment and Control on Chemical Process, School of Resources and Environmental Engineering, East China University of Science and Technology, Shanghai, People’s Republic of China; Nanjing Agricultural University, CHINA

## Abstract

Species delimitation of *Psoroptes* spp. and identity of the parasite in water buffaloes remain poorly defined. In this study, *Psoroptes* infestation on three water buffalo farms in Egypt was examined based on morphometric characteristics, especially the opisthosomal setae of adult male mites. Clinical investigations showed that 28% (196/700) of the sampled animals had mange infestation. Microscopic examinations of 80 skin scrapings indicated the occurrence of *Psoroptes* mites in 17 (21.3%) samples, *Sarcoptes* mites in 27 (33.7%) samples, and the concurrence of both in 36 (45.0%) samples. Morphologically, the *Psoroptes* parasite was identified as *Psoroptes natalensis*. DNA sequence analysis of the second internal transcribed spacer (ITS2) in 11 representative samples confirmed the diagnosis and suggested the presence of a distinct variety of *Psoroptes natalensis* in Egypt.

## Introduction

The cosmopolitan *Psoroptes* mites (Acari: Psoroptidae) are important etiological agents of mange in domesticated and wild ungulates, resulting in significant economic losses and animal welfare concerns [1–3]. These astigmatid mites are obligatory ectoparasites on different body surfaces of the host, including the inner ears [4]. *Psoroptes* spp. are non-burrowing mites that feed superficially on lipid emulsion of the lymph, skin cells, and skin exudates by abrading the epidermis [5]. In addition, the presence of actively feeding mites may lead to inflammation, exudation of excess lymph, and crust formation on the skin [6–9]. Coproantigens of mites may cause hypersensitivities in affected hosts [10, 11]. These lead to hair loss, reduced weight gain and, in severe cases, death due to dehydration, pneumonia or bacterial septicaemia [12, 13].

Morphologically, *Psoroptes* mites have a relatively oval body that is slightly dorso-ventrally flattened. Morphometric characteristics form the basis of identification and classification of the genus *Psoroptes*, at least to the species level [14]. The genus is distinguished from others by the presence of a modified pretarsus consisting of a sucker-like ambulacral disk on a relatively long, 'segmented' ambulacral stalk [4, 15]. The outer opisthosomal setae of adult males have been commonly used to distinguish the five accepted species of the genus [16]. However, considerable variations in the length of outer opisthosomal setae have been observed within and between populations of mites, and these differences are not directly related to host species or geographical origins [16]. Thus, there is an ongoing debate on taxonomic validity of these species [17].

DNA sequence analysis has been used in the identification of *Psoroptes* spp. [3, 16, 18–20]. One of the molecular markers is the ribosomal internal transcribed spacer (ITS) of the rRNA gene unit [16]. This region appears to be well suited to studies of species groups [21, 22] as it is evolving at a higher rate than the coding regions of the rRNA genes [23].

Disease caused by *Psoroptes* mites is largely controlled or under control (mostly presented as subclinical infestations) in industrialized countries such as the United States and United Kingdom [8, 19, 24–26], although re-emergence in some areas is reported [11]. In developing countries, poor hygiene and husbandry practices have facilitated the spread of mange mites. A high infestation rate (83.2%) of mange mites (*Psoroptes* and *Sarcoptes*) has been reported in buffaloes (*Bubalus bubalis*) in Egypt [27].

Since its initial introduction into Egypt from India in the medieval, the water buffalo represents the main livestock asset of many Egyptians, especially smallholders, due to the high meat quality of the animal and high protein and fat contents of the milk. *Psoroptes* mange was detected as early as 1934 in Egyptian water buffaloes by Carpano [28], followed by several studies on the incidence and clinical manifestations of infestations [29–31]. Several previous studies reported the occurrence of *Psoroptes* sp. in buffaloes along with *Sarcoptes* sp. [27, 32] and *Chrioptes* sp. [33]. However, few studies have been conducted on molecular identification of mange mites in Egypt [34], especially *Psoroptes* spp. The present study was designed to determine the identity of *Psoroptes* mites in water buffaloes in Egypt using both morphological (light and scanning electron microscopy) and molecular (sequence analysis of the ITS2 region) tools.

## Material and Methods

### Ethics Statement

This study was carried out in compliance with the Guidelines of the Animal Health Research Institute, Egypt. The study protocol was approved by the Committee on the Ethics of Animal Health Research Institute, Egypt (Permit Number 32 approved on May 31, 2010). All scrapings were performed by licensed veterinarians from animals on private farms as part of routine clinical examinations and care, with written consents from the owners.

### Collection of samples

A total of 700 water buffaloes on three farms (A, B and C) in the Kafr El Sheikh District (within area of 5 square Km), Kafr El Sheikh (130 Km northeast of Cairo), Egypt were examined for the presence of mange mites during April to August 2011. Eighty individual skin scrapings were collected from mange-affected areas of animals, placed in plastic bags labelled with animal ear tag numbers, and transferred at ambient temperature to the laboratory. Skin scrapings in Petri dishes were screened by microscopy for the presence of live mites. *Psoroptes*-positive specimens were divided into two parts; the first was used in light microscopy and the other was fixed in 75% ethanol and stored at 4°C for molecular biologic analysis.

### Light microscopy

Mite specimens were cleared in Nesbitt’s fluid (40 g of chloral hydrate, 25 ml of distilled water 25 ml, and 2.5 ml of concentrate HCl) as described by Sanders et al. [4] for 3 days, and mounted in Hoyer’s medium (50 ml of distilled water, 30 g of gum Arabic, 200 g of chloral hydrate, and 20 ml of glycerin). Mounted specimens were examined under a light microscope equipped with an ocular micrometer and a camera Lucida. Morphometric determinations, based on measurements obtained from 10 samples, were made according to previous studies [35, 36]. The Grandjean system for the nomenclature (chaetotaxy) of setae was followed according to Bochkov [35] and Griffiths et al [37].

### Scanning Electron Microscopy (SEM)

The preparation of mite specimens for SEM analysis was performed according to the method of Panyarachun et al. [38]. Briefly, mites were fixed in 2.5% glutaraldehyde in 0.1 M phosphate buffer solution (pH 7.2) at 4°C for 3 h. Specimens were washed three times with the buffer, and dehydrated at 4°C through a graded series of ethanol. Mites were dried using the critical point method, mounted using carbon paste on Al-stub, and coated with gold up to the 400 Ǻ thickness in a sputter-coating unit (JFC-1100 E; JEOL Ltd, Tokyo, Japan). The specimen was examined in a JEOL JSM-5300 (JEOL Ltd, Tokyo, Japan) scanning electron microscope operating at 25 keV.

### DNA extraction and PCR amplification

Eleven representative *Psoroptes*-positive skin scrapings were washed with distilled water by centrifugation. DNA was extracted from the specimens using the FastDNA SPIN Kit for Soil (MP Biomedicals, Colon, OH). PCR amplification of the ITS2 was done using primers RIB-4 and RIB-3 as described by Zahler et al. [18].

### DNA sequence analyses

PCR products were sequenced in both directions using the Big Dye® Terminator v3.1 Cycle Sequencing Kit (Applied Biosystems, Foster City, CA) and an ABI 3130 Genetic Analyzer (Applied Biosystems). The bi-directional sequences were assembled using the ChromasPro (version 1.5) software (http://www.technelysium.com.au/ChromasPro.html). The sequences obtained were aligned with each other and reference sequences from GenBank using ClustalX (http://www.clustal.org/). A neighbor-joining (NJ) analysis implemented in the MEGA5 (http://www.megasoftware.net) was used to assess the phylogenetic relationship among some of the Sarcoptoidea mites (*Psoroptes* and *Chorioptes*), using Saitou and Nei distances and *Otodectes cynotis* (HQ728005) as the outgroup. Unique nucleotide sequences generated in this study were deposited in GenBank under accession numbers AB968081 to AB968091.

## Results

### Occurrence of *Psoroptes* sp.

Clinical examinations indicated that 196 of the 700 (28%) water buffaloes had mite infestations characterized by hair loss, crust formation and pruritic dermatitis. There were no obvious differences in the infestation rates among the three farms (A: 91/313; B: 41/157; C: 64/230). The infestation occurred mainly in the perineal region, back, shoulders, withers and neck of animals. Microscopic investigation of skin scrapings from of 80 specimens showed that 27 (33.75%) had *Sarcoptes* sp., 17 (21.25%) had *Psoroptes* sp., whereas 36 (45%) had mixed infestations with both. Skin scrapings with mixed infestations were all collected from the perineal region at the base of the tail.

### Morphometric description of *Psoroptes* mites

**Fig 1:**
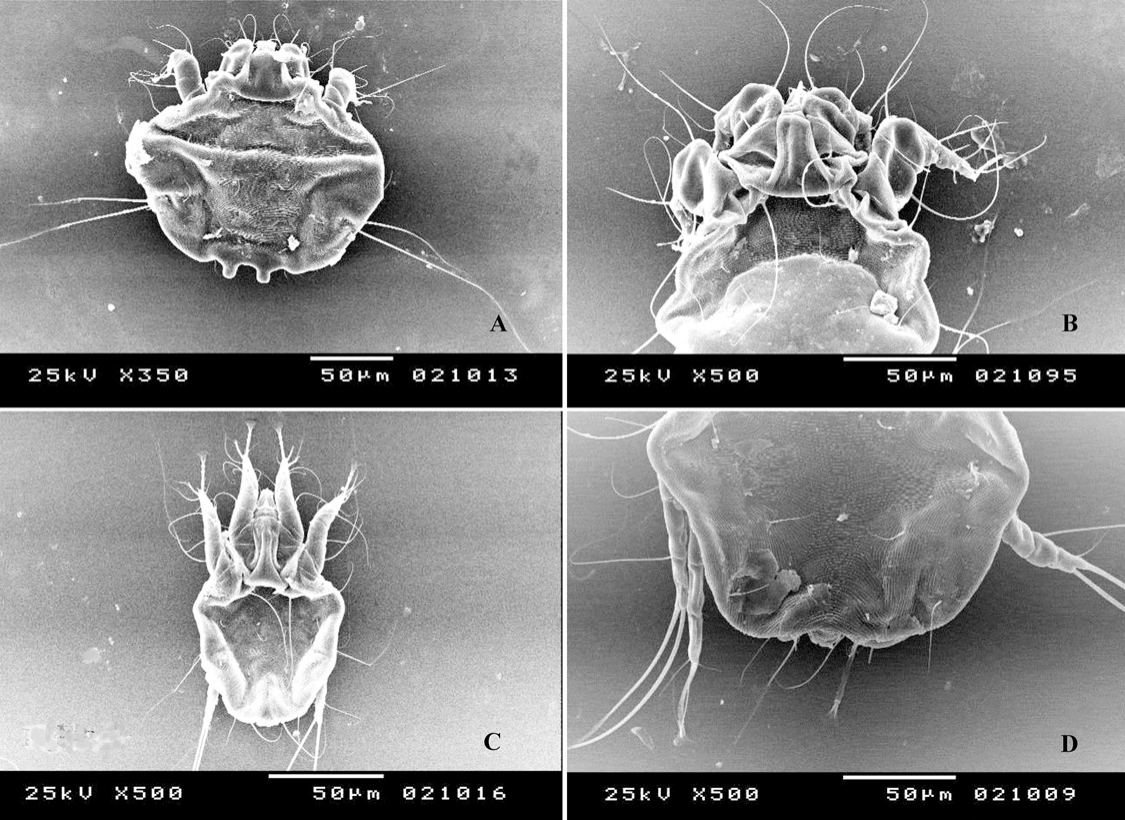
Scanning electron microscopy images of an adult male *Psoroptes natalensis* mite (A), showing the propodosomal and hysteronotal shields (B) as well as an adult female mite (C), showing the opisthosomal region (D).

**Fig 2:**
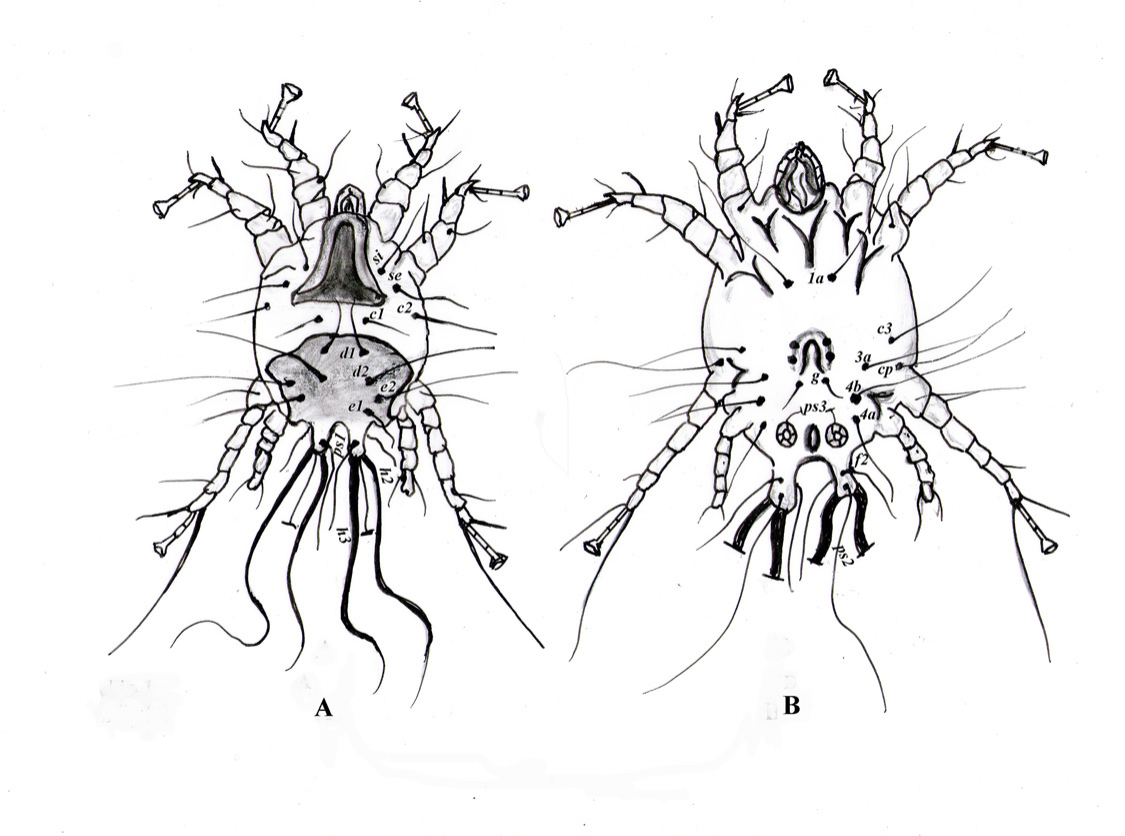
Illustration of a male *Psoroptes natalensis* mite showing body configuration and distribution of body appendages and setae. **A:** dorsal surface showing the propodosomal and hysteronotal shields and propodosomal (*si* and *se*), metapodosomal (*c1* and *c2*) and hysteronotal setae (*d1*, *d2*, *e1*, *e2* and *ps1*). Outer opisthosomal setae (*h2*) and the inner one (*h3*) were spatulated. B: ventral surface showing the aedeagus located in the metapodosomal region, along with a pair of lateral cuticular pits, followed posteriorly with a pair of adanal suckers, propodosomal setae (*1a*), and metapodosomal setae (*c3*, *cp*, *4b*, *3a* and *4a*).

Dorsally, male mites showed a relatively oval body of ~336 μm (270–343 μm) in length and ~307 μm (270–335 μm) in width, including the gnathosoma and opisthosomal lobes (Fig 1A). The gnathosoma, measured ~80 μm (71–98 μm) in length and ~47 μm (43–59 μm) in width, was protruding anteriorly. The idiosoma measured ~ 257 μm (197–300 μm) and had well sclerotized propodonotal and hysteronotal shields (Figs 1B and 2A). Except for the propodonotal and hysteronotal plates, the dorsal surface was decked with fine transverse, nearly parallel striations. Legs I, II and III carried a modified pretarsus consisting of a sucker-like ambulacral disk on a relatively long, 'segmented' ambulacral stalk. A total of nine pairs of setae were seen on the dorsal surface (Fig 2A). Thus, two pairs of setae (*si* and *se*), could be seen in the propodosomal plate, whereas two pairs of metapodosomal setae (*c1* and *c2*) and five pairs of hysteronotal setae (*d1*, *d2*, *e1*, *e2* and *ps1*) were seen in the idiosoma, with the last one situated on the opisthosomal lobe. Each opisthosomal lobe further had three long (*h2*, *ps2*, *h3*) and two (*ps1* and *f2*) short setae. The outer opisthosomal (*h2*) and inner (*h3*) setae were spatulated and of almost equal length; 231 (210–260) and 221 (198–270) μm, respectively.

Ventrally, the aedeagus of the adult male mite was located on the metapodosomal region, along with a pair of lateral cuticular pits, followed posteriorly with a pair of adanal suckers (Fig 2B). Seven pairs of setae could be recognized, including one pair of propodosomal setae (*1a*) between the coxa of the first and second pair of legs, five pairs of idiosomal setae in the metapodosomal region of the coxa of the 3^rd^ and 4^th^ pair of legs (*c3*, *cp*, *4b*, *3a* and *4a*), one pair of short setae behind the aedeagus (*g*), and one pair of short adanal setae (*ps3*) located slightly anterior and lateral to the anus.

**Fig 3:**
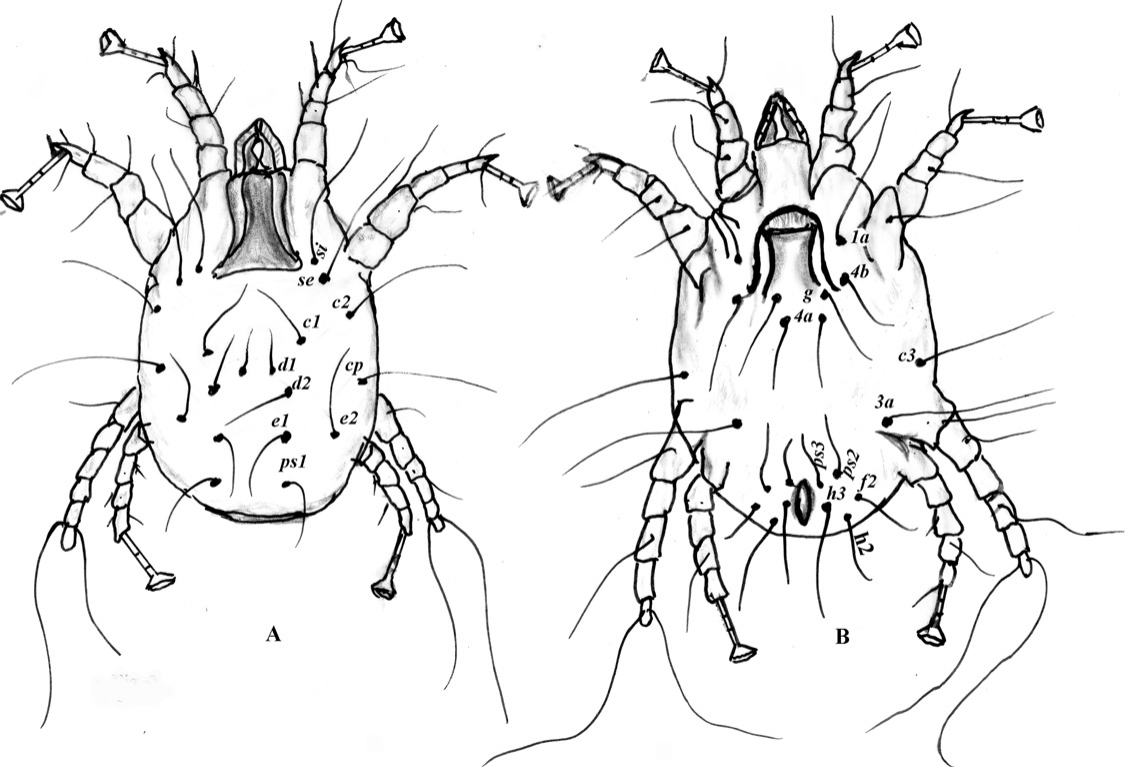
Illustration of a female *Psoroptes natalensis* mite showing body configuration and distribution of body appendages and setae. **A:** dorsal surface showing the propodosomal plate and propodosomal (*si* and *se*), metapodosomal (*c1*, *c2*, *cp*, *d1* and *d2*) and opisthosomal setae (*e1*, *e2* and *ps1*). B: ventral surface showing the vulva as a transverse slit with two lateral limbs and supported with three pairs of setae; two lateral pairs (*4b* and *g*) and one pair behind the vulvar flap (*4a*), propodosomal setae (*1a*), metapodosomal setae (*3a* and *3c*) and five pairs of perianal setae (*ps2*, *ps3*, *h2*, *h3* and *f2*) around the subterminal anus.

The adult female was larger than the adult male, measuring about 508 (465–600) μm in length and 381(355–425) μm in width, including the gnathosoma [~ 113 μm (87–125) and ~ 72 μm (61–93) in length and width, respectively] and idiosoma [~ 398 μm (375 and ~417 μm)]. Whereas the propodonotal plate was well developed, the hysteronotal shield was absent (Figs 1C, 1D and 3A), compared with the adult male. Except for propodonotal plate, the dorsal surface was decked with fine transverse striations (Fig 1C). The opisthosoma had a blunt posterior margin (Fig 1C and 1D). Legs I, II and IV carried a modified pretarsus consisting of a sucker-like ambulacral disk on a relatively long, 'segmented' ambulacral stalk (Fig 1D). The adult female had 10 pairs of setae on its dorsal surface (Fig 3A), including two pairs in the propodosomal region (*si* and *se*) and eight pairs in the idiosomal region. The idiosomal setae included five pairs of metapodosomal (*c1*, *c2*, *cp*, *d1* and *d2*) and three pairs of opisthosomal setae (*e1*, *e2* and *ps1*).

Ventrally, the adult female had a vulva along with two pairs of cuticular pits (genital suckers) in the propodosomal region, and a subterminal anus at the posterior margin of the body. The ventral surface of the female was nearly flat and finely striated, with the striations forming a triangular area above the vulva (Fig 3B). The vulva had a transverse slit with two lateral limbs and was supported with three pairs of setae; two lateral pairs (*4b* and *g*) and one pair behind the vulvar flap (*4a*). In addition, there were one pair of the propodosomal setae (*1a*) between the coxa of the first and second pairs of legs, two pairs of idiosomal setae (*3a* and *3c*) in the metapodosomal region and five pairs of perianal setae (*ps2*, *ps3*, *h2*, *h3* and *f2*) around the anus.

### Molecular characterization of *Psoroptes* sp.

Sequence analysis of 11 representative specimens from the three farms generated two types of ITS-2 sequences. The first type was represented by nine identical sequences and had considerable differences (4–9%) from those in GenBank, including nucleotide substitutions, insertions and deletions. The other sequence type was represented by two sequences (AB968084 from Farm A and AB968091 from Farm B) and had nucleotide substitutions of C to T at position 23, T to A at position 28, C to A at position 33, A to G at position 170, and G to A at position 248 compared to the first sequence type.

**Fig 4:**
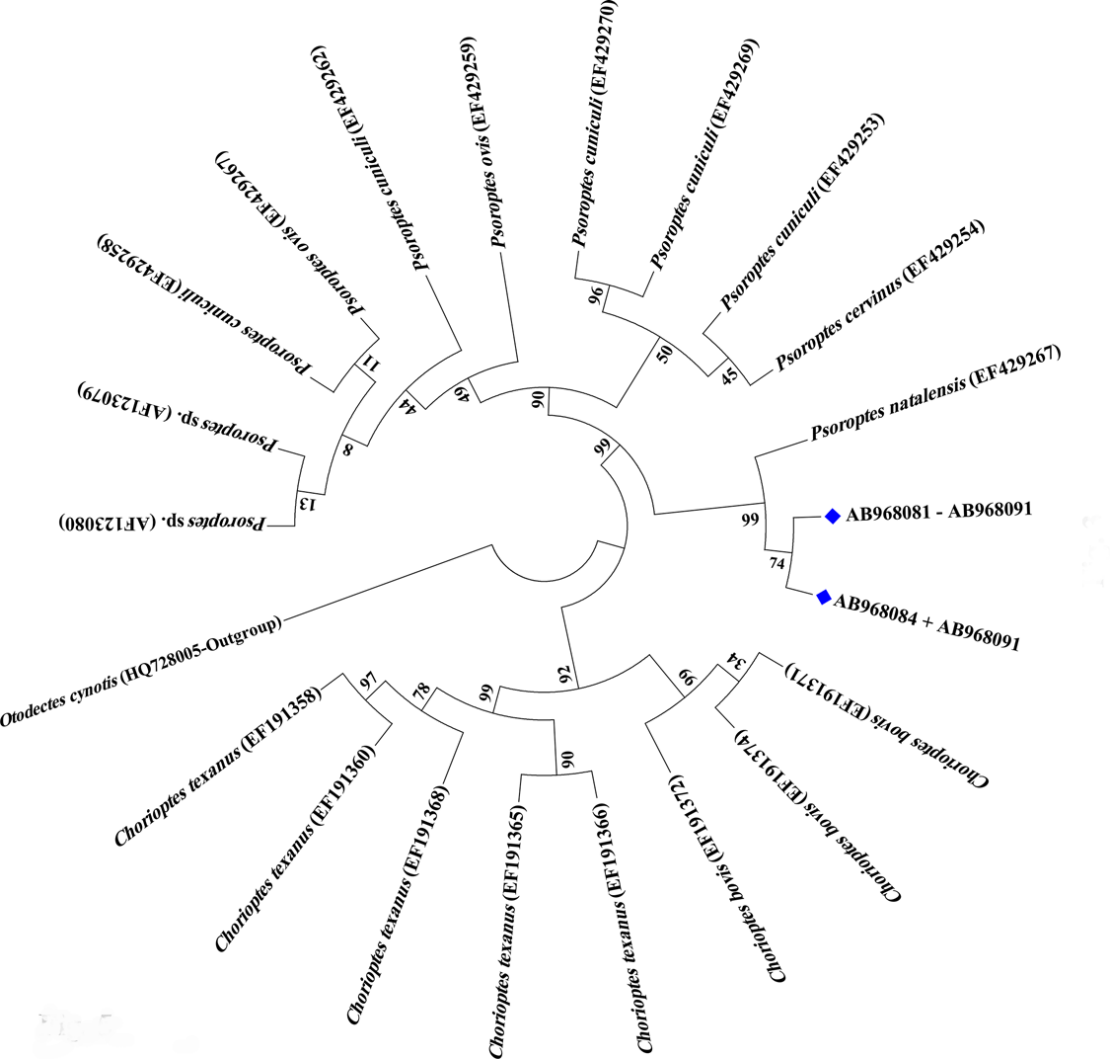
Phylogenetic relationship of *Psoroptes* spp. based on partial sequences of the ITS2. Evolutionary relationships of 22 taxa were inferred using the Neighbor-Joining method and Saitou and Nei distances, with the *Otodectes cynotis* (HQ728005) as the outgroup. Numbers at the internodes correspond to percent bootstrap values from 5,000 replicates.

In a NJ analysis, all *Psoroptes* ITS2 sequences from this study formed a monophyletic group, and clustered with the only available sequence (EF025929) of *Psoroptes natalensis* isolated from buffaloes in China (Fig 4). The *P*. *natalensis* branch was clearly separated from other *Psoroptes* taxa with 92% bootstrap support. However, there was no clear separation among other *Psoroptes* populations referred to as *P*. *cuniculi*, *P*. *ovis*, and *P*. *cervinus* (Fig 4).

## Discussion

A common occurrence of *Psoroptes* mites was seen in water buffaloes in this study. Although the 28% infestation rate of mange is much lower than that reported (~82%) by Yassin [27] in water buffaloes in the Giza Governorate, it is higher than that reported (16.66%) by El-Khodery et al. [39] in water buffaloes in the Nile Delta region. Elsewhere, Kazmi et al. [40] reported that ~11.0% of buffaloes in Lahore, Pakistan had mange infestation. Such differences might be attributed to differences in geographic locations, seasons of sample collection, breeds of the animals, and general hygiene and animal management. Results of the present study indicated that 45% of the infected animals had mixed infestations of both *Sarcoptes* and *Psoroptes* mites. These findings are in agreement with earlier observations in Egypt [27, 32]. Mixed *Sarcoptes* and *Psoroptes* infestations have also been reported in water buffaloes in India [41, 42]. In addition, El-Khodery et al. [33] reported mixed infestations of *Psoroptes* sp. and *Chrioptes* sp. in water buffaloes in Egypt. However, this is the first report on the occurrence of both *Sarcoptes* and *Psoroptes* mites at the same predilection site. Burrowing *Sarcoptes* spp. may provide nutrients to non-burrowing *Psoroptes* spp. by increasing the availability of lymph and tissue exudates. Nevertheless, a considerable fraction (21.3%) of the mange infestation was attributed to *Psoroptes* mites alone, although it is lower than that reported by El-Khodery et al. [33] in water buffaloes in the Nile Delta region, Egypt.

Morphologically, all *Psoroptes* mites showed the presence of a terminal sucker on a relatively long jointed pre-tarsus, which is a distinctive feature of the genus *Psoroptes* [4, 15, 31, 35]. The outer (*h2*) and inner (*h3*) opisthosomal setae were spatulated and of almost equal length, which are distinctive features of *P*. *natalensis* [15, 29, 31, 43]. The small morphometric differences reported in the present study compared to earlier ones [16, 17] may likely be the result of phenotypic adaptation to microenvironments [16].

Sequence and phylogenetic analysis based on the ITS2 locus supported the morphologic identification of *P*. *natalensis*. This is in agreement with the findings by Wang et al [44] who reported that phylogenetic analysis of the 18S rRNA and COX1 genes could reliably differentiate *P*. *natalensis* from *P*. *cuniculi*. In contrast, we could not identify any monophyletic groups formed by other *Psoroptes* species based on sequence analysis of ITS2. Similarly, Pegler et al. [16], Zahler et al. [18], Ramey et al. [19] and Bates [45] could not find any genetic (based on ITS1 and ITS2) support for the morphologically differentiated *P*. *cuniculi*, *P*. *ovis* and *P*. *cervinus*.

Notably, there are obvious sequence differences (4–9%) in ITS2 sequences between this and previous studies. It is not known whether this sequence heterogeneity is due to recent gene flow or genetic recombination [19]. Experimentally, cross mating between different nominal species of *Psoroptes* mites is possible, producing viable offspring [4, 46]. Although paralogous ITS sequences are common in the mite genome [19], this appears not likely to be the case in the present study, as all specimens from different farms except two produced identical sequences. It is possible that the rRNA repeat units may be homogenized through the process of concerted evolution or unequal crossing-over. In addition, host factors may play an important role in shaping the genetic structure of the parasitizing mites. Ochs et al. [47] reported that ITS2 sequences of *Chorioptes* isolates from sheep and a camel differed by 18% from the sequence of an isolate from a cow, suggesting the existence of epidemiologically segregated populations. Being the ancient land bridge between Africa and Asia, Egypt has extensive movement of livestock, pets and wild animals, which might facilitate the gene flow among divergent psoroptic mite populations. Further studies are needed to determine whether the *P*. *natalensis* variant identified in this study is unique to water buffaloes in Egypt.
